# Examining the effector mechanisms of Xuebijing injection on COVID-19 based on network pharmacology

**DOI:** 10.1186/s13040-020-00227-6

**Published:** 2020-10-16

**Authors:** Wen-jiang Zheng, Qian Yan, Yong-shi Ni, Shao-feng Zhan, Liu-liu Yang, Hong-fa Zhuang, Xiao-hong Liu, Yong Jiang

**Affiliations:** 1 The First Clinical Medical School of Guangzhou University of Chinese Medicine, Guangzhou, China; 2 The Second Clinical Medical School of Guangzhou University of Chinese Medicine, Guangzhou, China; 3 The First Affiliated Hospital of Guangzhou University of Chinese Medicine, Guangzhou, China; 4 Shenzhen Hospital of Integrated Traditional Chinese and Western Medicine, Shenzhen, China

**Keywords:** Active ingredient, Coronavirus disease 2019, Effector mechanism, Molecular docking, Network pharmacology, Xuebijing

## Abstract

**Background:**

Chinese medicine Xuebijing (XBJ) has proven to be effective in the treatment of mild coronavirus disease 2019 (COVID-19) cases. But the bioactive compounds and potential mechanisms of XBJ for COVID-19 prevention and treatment are unclear. This study aimed to examine the potential effector mechanisms of XBJ on COVID-19 based on network pharmacology.

**Methods:**

We searched Chinese and international papers to obtain the active ingredients of XBJ. Then, we compiled COVID-19 disease targets from the GeneCards gene database and via literature searches. Next, we used the SwissTargetPrediction database to predict XBJ’s effector targets and map them to the abovementioned COVID-19 disease targets in order to obtain potential therapeutic targets of XBJ. Cytoscape software version 3.7.0 was used to construct a “XBJ active-compound-potential-effector target” network and protein-protein interaction (PPI) network, and then to carry out network topology analysis of potential targets. We used the ClueGO and CluePedia plugins in Cytoscape to conduct gene ontology (GO) biological process (BP) analysis and Kyoto Encyclopedia of Genes and Genomes (KEGG) signaling pathway enrichment analysis of XBJ’s effector targets. We used AutoDock vina and PyMOL software for molecular docking.

**Results:**

We obtained 144 potential COVID-19 effector targets of XBJ. Fourteen of these targets-glyceraldehyde 3-phosphate dehydrogenase (*GAPDH*), albumin (*ALB*), tumor necrosis factor (*TNF*), epidermal growth factor receptor (*EGFR*), mitogen-activated protein kinase 1 (*MAPK1*), Caspase-3 (*CASP3*), signal transducer and activator of transcription 3 (*STAT3*), *MAPK8*, prostaglandin-endoperoxide synthase 2 (*PTGS2*), *JUN*, interleukin-2 (*IL-2*), estrogen receptor 1 (*ESR1*), and *MAPK14* had degree values > 40 and therefore could be considered key targets. They participated in extracellular signal–regulated kinase 1 and 2 (*ERK1*, *ERK2*) cascade, the T-cell receptor signaling pathway, activation of *MAPK* activity, cellular response to lipopolysaccharide, and other inflammation- and immune-related BPs. XBJ exerted its therapeutic effects through the renin-angiotensin system (RAS), nuclear factor κ-light-chain-enhancer of activated B cells (NF-κB), *MAPK*, phosphatidylinositol-4, 5-bisphosphate 3-kinase (*PI3K*)-protein kinase B (*Akt*)-vascular endothelial growth factor (VEGF), toll-like receptor (TLR), TNF, and inflammatory-mediator regulation of transient receptor potential (TRP) signaling pathways to ultimately construct a “drug-ingredient-target-pathway” effector network. The molecular docking results showed that the core 18 effective ingredients had a docking score of less than − 4.0 with those top 10 targets.

**Conclusion:**

The active ingredients of XBJ regulated different genes, acted on different pathways, and synergistically produced anti-inflammatory and immune-regulatory effects, which fully demonstrated the synergistic effects of different components on multiple targets and pathways. Our study demonstrated that key ingredients and their targets have potential binding activity, the existing studies on the pharmacological mechanisms of XBJ in the treatment of sepsis and severe pneumonia, could explain the effector mechanism of XBJ in COVID-19 treatment, and those provided a preliminary examination of the potential effector mechanism in this disease.

## Introduction

In December 2019, the Huanan Seafood Wholesale Market in Wuhan, Hubei Province, China, became the epicenter of an outbreak of a pneumonia of unknown etiology, which attracted a great deal of attention in China and the rest of the world. Chinese scientists quickly isolated a novel coronavirus from patients, severe acute respiratory syndrome coronavirus 2 (SARS-CoV-2), the causative agent of coronavirus disease 2019 (COVID-19) [1]. As of March 29, 2020, the global number of confirmed cases was 634,835 and the global death toll was 29,891. Currently, scientists around the world are conducting an exhaustive search for effective antiviral drugs. However, the only feasible method at this writing is the use of various broad-spectrum antivirals, such as nucleoside analogs and human immunodeficiency virus (HIV) protease inhibitors [2]. Up to now, no COVID-19 specific antiviral drugs or vaccines have been developed, and the aforementioned drugs can only reduce viral infection [3].

Many results from clinical practice show that TCM plays an important role in COVID-19 treatment and has brought new hope for controlling this disease [4]. Xuebijing (XBJ) was included in the *Diagnosis and Treatment Plan for Coronavirus Disease 2019* (interim 7th edition) that was jointly released by the National Health Commission and National Administration of Traditional Chinese Medicine. XBJ is composed of extracts of *Carthamus tinctorius*, *Paeonia anomala*, *Ligusticum striatum*, *Salvia miltiorrhiza*, and *Angelica sinensis* [5]. This compound can boost circulation, relieve stasis, and clear blocked meridians, and it is widely used in China to treat active inflammation [6]. In 2004, XBJ was approved by the National Medical Products Administration for the treatment of systemic inflammatory-response syndrome, sepsis, and multiple-organ dysfunction syndrome (MODS) [7, 8]. Studies show that XBJ treatment can reduce the secretion of pro-inflammatory cytokines such as interleukin (IL)-6, IL-13, and tumor necrosis factor alpha (TNF-α) to alleviate inflammation and thereby inhibit liver damage [9]. Chen et al. showed that XBJ treatment can decrease oxidative stress (OS) and levels of pro-inflammatory cytokines [10]. Li et al. showed that XBJ can regulate immune response, including reducing inflammatory mediators and bacterial load, and plays a protective role in bacterial infections, particularly those caused by drug-resistant bacteria such as methicillin-resistant *Staphylococcus aureus* (MRSA) [11]. A real-world study also pointed out that the incidence of adverse reactions from XBJ in clinical practice is low (0.3%), and most adverse reactions are mild. Therefore, XBJ could be a safe and effective drug for treating COVID-19, but its specific molecular mechanisms are still unknown.

Network pharmacology (NP) uses drug, compound, gene, and disease database information to construct drug-target, target-disease, and drug-disease interaction networks in order to reveal the complex mechanisms of TCM formulations that have multiple targets and multiple component characteristics [12]. The concepts of NP share many similarities with the holistic view of TCM, as both use systemic methods to treat complex diseases such as cancer. This provides a basis for the transition from empirical medicine to evidence-based medicine [13].

Therefore, we employ NP to initially explore the potential molecular mechanism of XBJ on COVID-19. The basis for our NP study rests on the notions that the proteins that associate with and functionally govern viral infection are localized in the corresponding subnetwork within the comprehensive human interactome network. The latest research shows that host cell pathways regulated by SARS-CoV-2 infection and showed that inhibiting these pathways can prevent virus replication in human cells [14]. A drug with multiple targets, such as XBJ, if it is to be effective against an HCoV, its direct or indirect target proteins should be within or related to the corresponding subnetwork in the human protein-protein interactome.

In terms of potential therapeutic targets, research shows that the 2019-nCoV/SARS-CoV-2 shares the highest nucleotide sequence identity (79.7%) with SARS-CoV among the six other known pathogenic HCoVs [15]. Full-length genome sequences from five patients at an early stage of COVID-19 outbreak showed that the sequences are almost identical and share a 79.6% sequence identity to SARS-CoV, what’s more, 2019-nCoV is 96% identical at the whole-genome level to a bat coronavirus [16], and the amino acid sequences and predicted protein structures of the receptor-binding domain (RBD) of SARS-CoV2 and SARS-CoV share a high similarities [17], all of these indicate that the host proteins of SARS-CoV were considered potential therapeutic targets for COVID-19.

In addition, viruses including coronaviruses like COVID-19 require host cellular factors for successful replication during infection. Study on virus-host protein-protein interactions (PPIs) provides an effective method toward elucidating the mechanisms of viral infection [18, 19], so the virus-host interactome may offer a strategy for the treatment of viral infections, and the human pathogenic coronaviruses SARS-CoV2, SARS-CoV, and Middle East respiratory syndrome coronavirus (MERS-CoV) belong to the family Coronaviridae and the genus Betacoronavirus, with similar infectivity, pathogenicity, and related clinical characteristics. Therefore, in our study, the host proteins of the relevant coronaviruses are considered as potential therapeutic targets for COVID-19. As well as angiotensin-converting enzyme 2 (ACE2) and coronavirus pneumonia related targets.

**Fig. 1:**
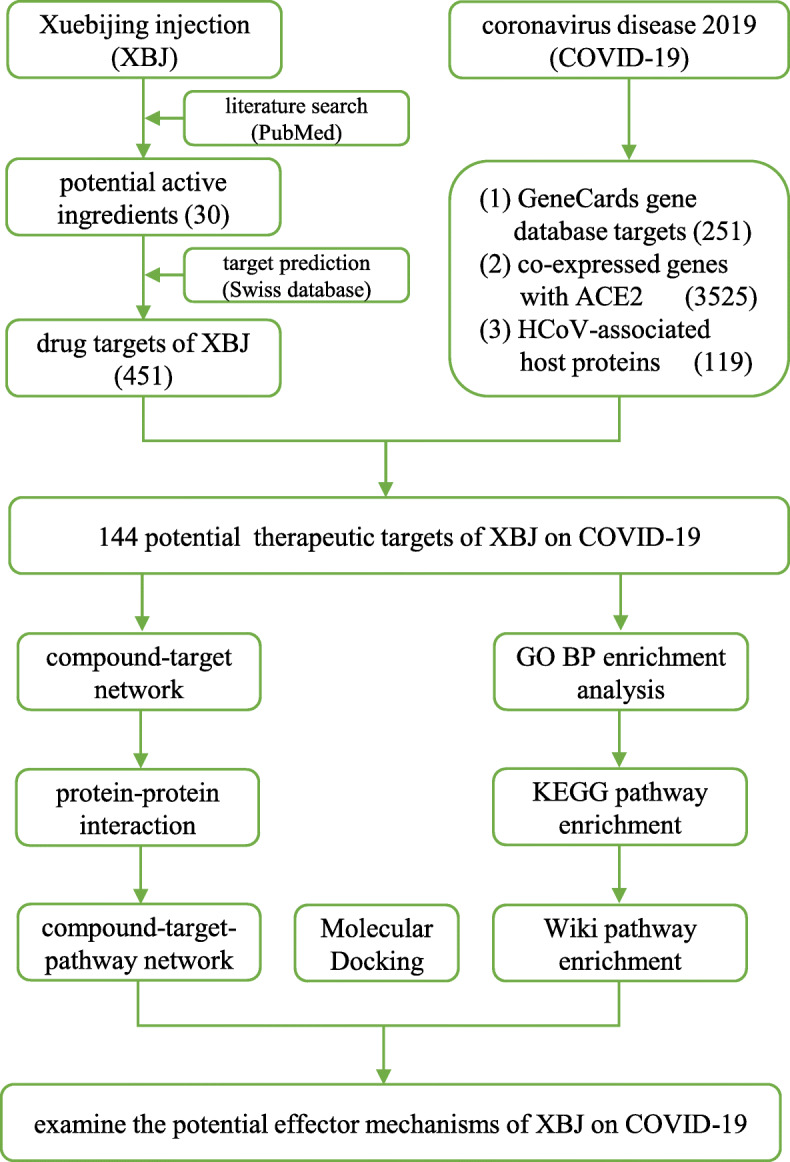
Overall workflow of this study

It is essential to integrate drug-target networks, HCoV-host interactions and human protein-protein interactome network. Based on chemical-matteromics study results for XBJ, we employed NP to construct a “component-target-pathway” network model in order to comprehensively and systematically predict potential effector targets and pathways of this compound’s main chemical components in COVID-19 treatment. These findings will provide a scientific basis for further research into effective substances and mechanisms in XBJ treatment of COVID-19, the overall workflow of this study was presented in Fig. 1.

## Materials and methods

### Collection of potential active ingredients of XBJ

We searched the global scientific literature to determine the active ingredients of XBJ. The PubChem (https://pubchem.ncbi.nlm.nih.gov/, National Center for Biotechnology Information, 8600 Rockville Pike, Bethesda, MD, USA) was used for cross-validation and to acquire the molecular structures of potential active chemical components, which we stored in canonical simplified molecular-input line-entry system (SMILES) format.

### Prediction of potential effector targets of XBJ

To predict potential effector targets, we used the SwissTargetPrediction database [20] (STP, http://www.swisstargetprediction.ch, version 2019, designed by Swiss Institute of Bioinformatics, Quartier Sorge - Batiment Amphipole 1015 Lausanne, Switzerland). We input the aforementioned potential active ingredients in SMILES format into this database, with “humans” (*Homo sapiens*) as the study species, to obtain the potential effector targets of the component compounds; results were stored in csv format. After compilation and removal of repetitions, we obtained the potential effector targets of XBJ.

### Screening of potential therapeutic targets of COVID-19

Three sources were used to obtain potential therapeutic targets of COVID-19. First, we obtained the COVID-19 disease target set by searching the GeneCards gene database (http://www.genecards.org, designed by The Weizmann Institute of Science, 234 Herzl Street, POB 26, Rehovot, Israel) [21] on the phrase “coronavirus pneumonia”. Second, we searched the literature to collect potential therapeutic targets of COVID-19. ACE is reported to be the receptor for SARS-CoV [22] and is also believed to be that for SARS-CoV-2. We used single-cell sequencing results for colon epithelial cells [23] to extract genes that are co-expressed with ACE2, which we matched with human targets as potential therapeutic targets for COVID-19. Third, we downloaded human coronavirus (HCoV)-related host proteins from the appendices of one study [15]. Specifically, these host proteins are either the direct targets of HCoV proteins or are involved in crucial pathways of HCoV infection. By regulating the relevant coronavirus host protein could lead to a therapeutic regimen to treat COVID-19 [24].The relevant coronaviruses include SARS-CoV, MERS-CoV, infectious-bronchitis virus (IBV), mouse hepatitis virus (MHV), HCoV-229E, and HCoV-NL63; the host proteins were considered potential therapeutic targets for COVID-19. Finally, we obtained intersections between the active-ingredient-related targets and the disease-related targets from the three abovementioned sources. Intersectional targets were considered potential therapeutic targets of XBJ in COVID-19.

### Construction of a network of active ingredients and effector targets

We input the abovementioned potential active compounds of XBJ and their potential effector targets into Cytoscape software (http://www.cytoscape.org, Version 3.7.0, designed by Department of Bioengineering, University of California-San Diego, California, USA) [25] to plot a “XBJ active-compound-potential effector target” network analysis map. On this map, different nodes represented potential active compounds and effector targets of XBJ, and the map’s edges showed relationships between these two factors.

### Protein-protein interaction (PPI) analysis and network topology analysis

We input the potential therapeutic targets of XBJ into the Search Tool for the Retrieval of Interacting Genes/Proteins (STRING, https://string-db.org/, supported by ELIXIR, Wellcome Genome Campus, Hinxton, Cambridgeshire, UK) [26] and selected humans as the species to obtain PPI data. Next, we input this data into Cytoscape to plot a PPI network map. The cytoHubba (Predicts and explores important nodes and subnetworks in a given network by several topological algorithms, provided by Institute of Information Science, Academia Sinica, Taiwan) [27] plugin in Cytoscape was used for network topology analysis, and results were sorted by the degree value.

### Gene functional annotation of potential effector targets

The org.Hs.eg.db (https://www.bioconductor.org/, a package for the genome wide annotation of Human, primarily based on mapping using Entrez Gene identifiers, version 3.1.1) and the clusterProfiler (https://www.bioconductor.org, a package implements methods to analyze and visualize functional profiles of gene and gene clusters, version 3.1.1) [28] on the R 3.5.2, were used to perform the gene ontology (GO) biological process (BP) enrichment analysis [29] and the Kyoto Gene and Genome Encyclopedia (KEGG) enrichment analysis [30] of the potential effector targets, and *P* < 0.05 were selected. We used the ClueGO (version 2.5.4, creates and visualizes a functionally grouped network of terms/pathways) [31] and CluePedia (version 1.5.4, a ClueGO plugin for pathway insights using integrated experimental and in silico data) (both provided by Laboratory of Integrative Cancer Immunology, Paris, France) [32] plugins in Cytoscape, to perform WikiPathways enrichment analysis of the potential effector targets. We chose the analysis mode of Functional analysis, load marker list is *Homo sapiens* (9606) and Symbol ID was input, then we selected “WikiPathways-503 terms/pathways with 6558 available unique genes-27.02.2019”. Regarding statistical options, we employed two-sided hypergeometric test and bonferroni step down for *p* value correction, and *P* < 0.01 were selected.

### Analysis of the binding activity of ingredients with its targets by molecular docking

We performed molecular docking by using Auto Dock vina [33] (version 1.1.2, an open-source program for doing molecular docking designed and implemented by Dr. Oleg Trott in the Molecular Graphics Lab at The Scripps Research Institute) to verify the binding activity of the core ingredients of XBJ with its potential targets of COVID-19. The 3D structure of the ingredient was obtained in the PubChem and the core target protein in the Protein Data Bank (https://www.rcsb.org/). The screening criteria for target proteins were: (1) the organisms were *Homo sapiens*; (2) the analysis method was X-ray diffraction; (3) resolution < 2.5A; (4) the released date was as late as possible; (5) preference was given to those containing unique ligands. The ingredients and protein structures were processed by AutoDockTools (version 1.5.6). The combinations of best docking score were visualized by PyMOL.

## Results

### Network analysis of active ingredients and effector targets

**Fig. 2:**
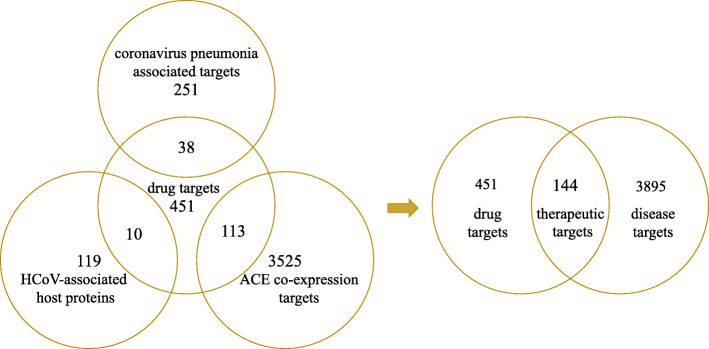
Schematic diagram of relationship between drug targets (XBJ) and disease (COVID-19-related) potential targets

**Fig. 3:**
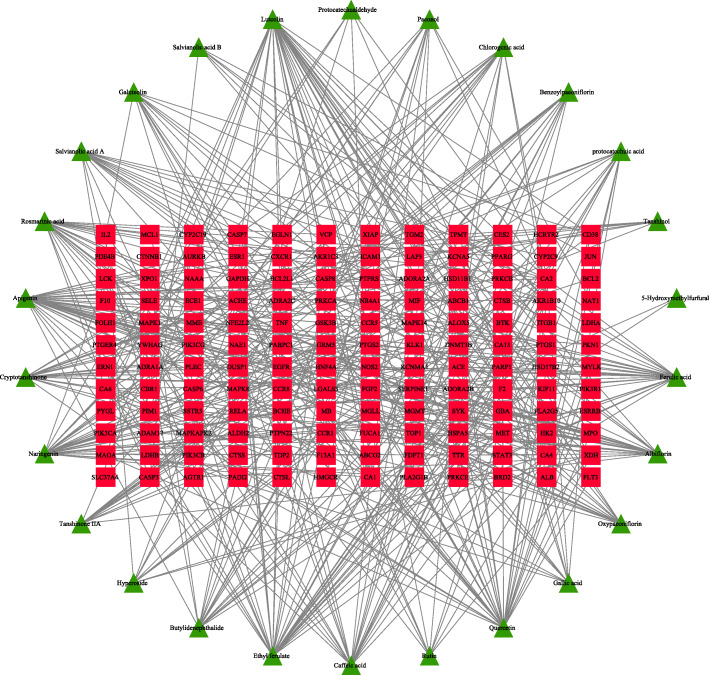
Potential active-ingredient potential therapeutic-target network analysis. Green triangles represent the 26 potential active ingredients of XBJ. Red squares represent 144 COVID-19-related potential effector targets

We selected 30 active ingredients of XBJ that had been detected by liquid chromatography-mass spectrometry (LC-MS) [34]. Results are shown in Table 1. 451 targets of XBJ for *Homo sapiens* were obtained based on Swiss database (The details of target information are shown in the Supplementary file, Table S1; 451 targets of XBJ are shown in Supplementary file, Table S2). We obtained 251 potential therapeutic targets of COVID-19 from the GeneCards database (Supplementary file, Table S3), 119 therapeutic targets were obtained from the systemic literature search (Supplementary file, Table S4) and Single-cell sequencing was used to obtain 3525 gene targets that were co-expressed with ACE (Supplementary file, Table S5). We obtained 144 potential COVID-19 therapeutic targets of XBJ in total after deduplication (Fig. 2 and Supplementary file, Table S6). We used Cytoscape to plot a “component-target” network relationship map (Fig. 3). Four ingredients had no targets that overlapped with potential therapeutic targets for COVID-19, so this network included 170 nodes, which were comprised of 26 XBJ components matched to 144 disease-related targets.

**Table 1:** Potential active ingredients of XBJ

No.	Compound	PubChem ID	Herbs
1	5-hydroxymethyl-furfural	237,332	Carthami Flos
2	Albiflorin	51,346,141	Radix Paeoniae Rubra
3	Apigenin	5,280,443	Radix Salviae, Carthami Flos
4	Benzoylpaeoniflorin	21,631,106	Radix Paeoniae Rubra
5	Butylidenephthalide	642,376	Chuanxiong Rhizoma, Angelicae Sinensis Radix
6	Caffeic acid	1,549,111	Chuanxiong Rhizoma
7	Catechinic acid	9064	Radix Paeoniae Rubra
8	Chlorogenic acid	1,794,427	Radix Salviae, Carthami Flos
9	Cryptotanshinone	160,254	Radix Salviae
10	Ethyl ferulate	736,681	Chuanxiong Rhizoma, Angelicae Sinensis Radix
11	Ferulic acid	445,858	Angelicae Sinensis Radix, Carthami Flos
12	Gallic acid	370	Radix Paeoniae Rubra
13	Galuteolin	5,317,471	Radix Salviae, Carthami Flos
14	Hydroxysafflor yellow A	49,798,103	Carthami Flos
15	Hyperoside	5,281,643	Carthami Flos
16	Luteolin	5,280,445	Radix Salviae, Carthami Flos
17	Naringenin	932	Carthami Flos
18	Oxypaeoniflorin	21,631,105	Radix Paeoniae Rubra
19	Paeonol	11,092	Radix Paeoniae Rubra
20	Protocatechuic acid	72	Radix Salviae
21	Protocatechuic aldehyde	8768	Radix Salviae
22	Quercetin	5,280,343	Carthami Flos
23	Rosmarinic acid	5,281,792	Radix Salviae
24	Rutin	5,280,805	Carthami Flos
25	salvianolic acid A	5,281,793	Radix Salviae
26	Salvianolic acid B	11,629,084	Radix Salviae
27	Senkyunolide I	11,521,428	Chuanxiong Rhizoma, Angelicae Sinensis Radix
28	Sodium Danshensu	23,711,819	Radix Salviae
29	Tanshinol	439,435	Radix Salviae
30	Tanshinone II A	164,676	Radix Salviae

### Network analysis of potential therapeutic targets

**Fig. 4:**
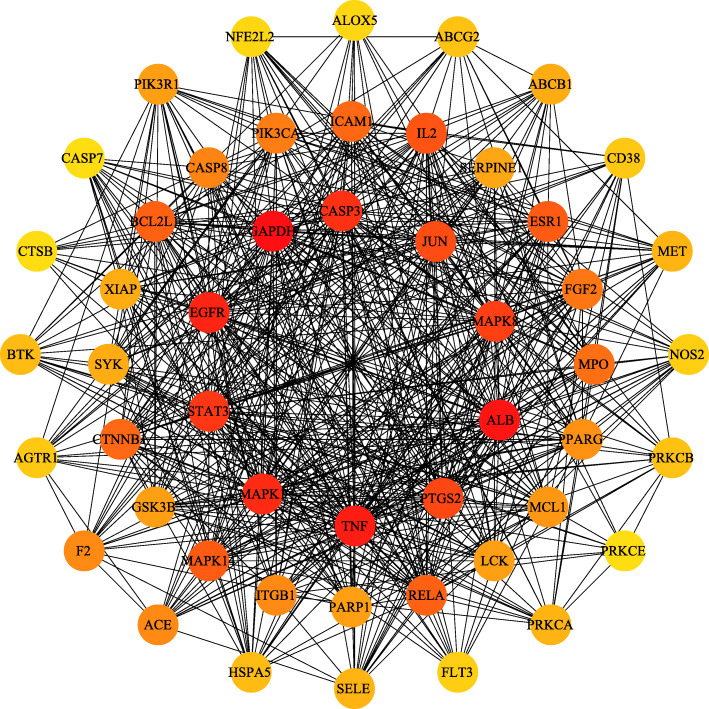
Network topology analysis. Black lines represent protein-protein interactions present. Deeper colors represent higher degree values

We input the PPI information obtained from STRING into Cytoscape to plot the PPI network, and then we used cytoHubba for network topology analysis. Table 2 shows the results, Fig. 4 shows the visualization results, and both show the top 50 potential therapeutic targets by degree values. The details of network topology analysis results of 114 potential therapeutic targets are shown in the Supplementary file, Table S7. Glyceraldehyde 3-phosphate dehydrogenase (*GAPDH*), *TNF*, mitogen-activated protein kinase 3 (*MAPK3*), Caspase-3 (*CASP3*), epidermal growth factor receptor (*EGFR*), *MAPK1*, prostaglandin-endoperoxide synthase 2 (*PTGS2*), signal transducer and activator of transcription 3 (*STAT3*), and *MAPK8* all had degree values > 40, showing that these proteins occupied important positions in the PPI network.

**Table 2:** Parameter information for network topology analysis of XBJ’s potential therapeutic targets

No.	Target	Degree	No.	Target	Degree
1	GAPDH	77	26	MCL1	30
2	ALB	73	27	LCK	27
3	TNF	70	28	PARP1	27
4	EGFR	63	29	GSK3B	27
5	MAPK1	61	30	PIK3R1	27
6	CASP3	58	31	SERPINE1	26
7	STAT3	55	32	ABCB1	25
8	MAPK8	49	33	SYK	25
9	PTGS2	48	34	XIAP	25
10	JUN	47	35	SELE	24
11	IL2	43	36	MET	24
12	ESR1	40	37	PRKCA	24
13	MAPK14	40	38	BTK	23
14	RELA	39	39	HSPA5	23
15	BCL2L1	39	40	ABCG2	22
16	ICAM1	38	41	PRKCB	22
17	CTNNB1	38	42	CD38	21
18	MPO	37	43	AGTR1	21
19	FGF2	35	44	FLT3	20
20	PIK3CA	34	45	NOS2	20
21	CASP8	33	46	NFE2L2	19
22	ACE	32	47	ALOX5	19
23	F2	32	48	CTSB	18
24	ITGB1	32	49	HNF4A	18
25	PPARG	31	50	PRKCE	18

### GO gene function and KEGG signaling pathway enrichment analyses

**Fig. 5:**
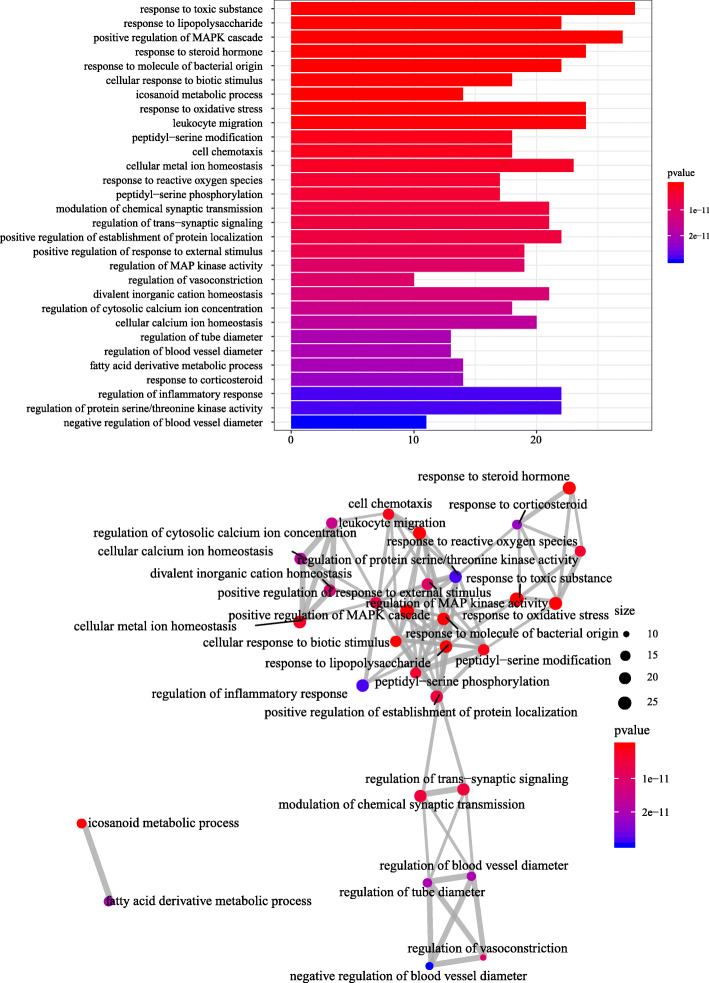
GO biological process enrichment analysis and network relationship

GO BP enrichment analysis showed that the potential therapeutic targets of XBJ involved 228 BPs. From those GO terms, it suggests that XBJ compounds may regulate the inflammatory response, vasoconstriction, and cytosolic calcium ion concentration; response to the corticosteroid, reactive oxygen species, oxidative stress, lipopolysaccharide, and biotic stimulus, and involve in cell chemotaxis, peptidyl-serine modification, leukocyte migration. This indicated that the active ingredients of XBJ could exert their effects through multiple BPs (Fig. 5 and Supplementary file, Table S8).

**Fig. 6:**
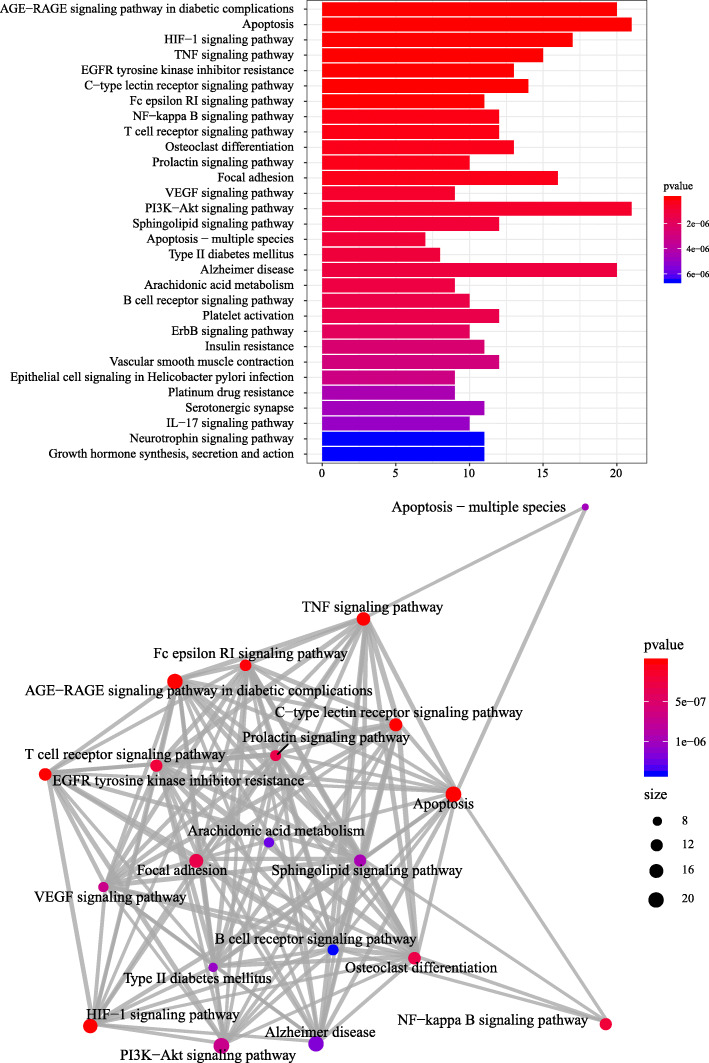
KEGG pathways enrichment analysis and network relationship

KEGG signaling pathway enrichment analysis showed that XBJ acted on COVID-19 through 94 pathways. From those KEGG terms, it shows that targets were significantly enriched in multiple pathways such as IL-17, B cell receptor, PI3K − Akt, VEGF, T cell receptor, NF-kappa B, TNF, HIF-1 signaling pathway, and vascular smooth muscle contraction, platelet activation, arachidonic acid metabolism, apoptosis. (Fig. 6 and Supplementary file, Table S9).

**Fig. 7:**
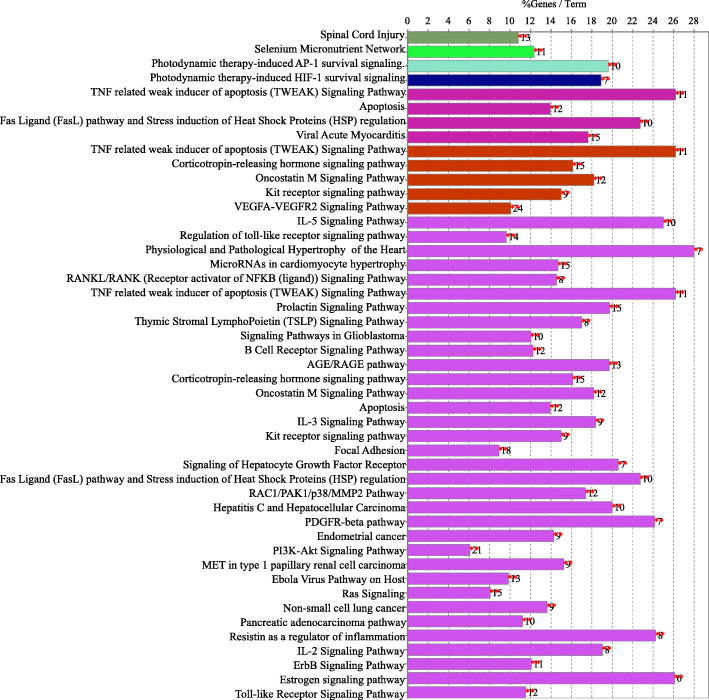
Pathways enrichment analysis based on WikiPathways database.**: *P*. values<0.001

WikiPathways enrichment analysis showed that XBJ acted on COVID-19 mainly through 40 pathways, such as HIF-1 survival, TWEAK, corticotropin-releasing hormone, RANKL/RANK, B Cell Receptor Signaling Pathway, and Ras Signaling Pathway. It also involved in some inflammatory signaling pathways like IL-2, IL-3, IL-5, PI3K-Akt, and Toll-like Receptor Signaling Pathway, and Ebola Virus pathway on host (Fig. 7 and Supplementary file, Table S10).

### Analysis of the ingredient-target interaction network and molecular docking

**Fig. 8:**
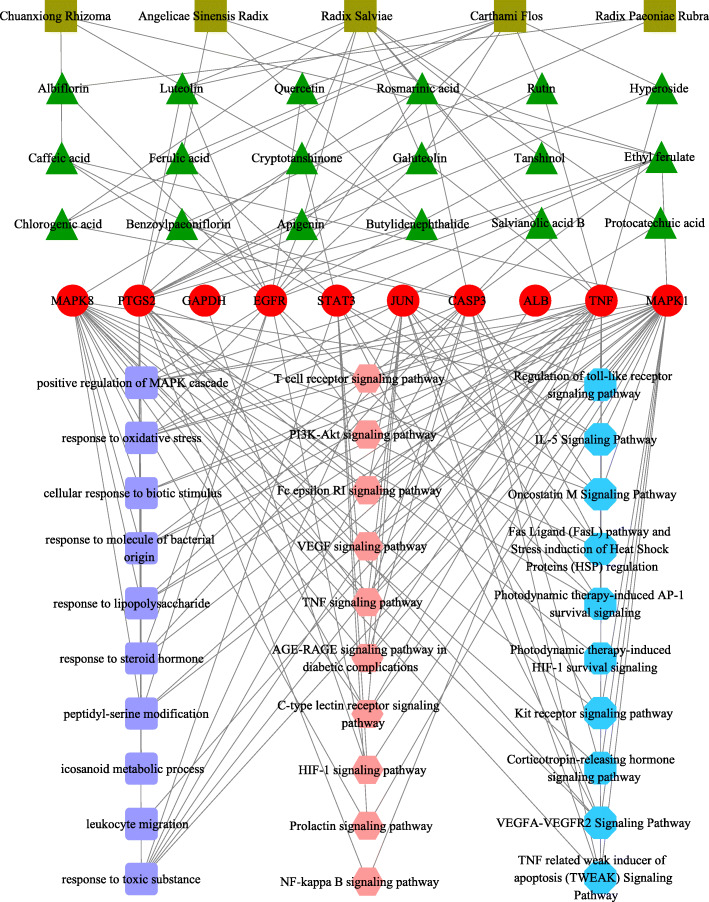
“Chinese herbal-active ingredient-effector target-biological process-signaling pathway” network

Based on the above analysis, we constructed an “ingredient-target interaction” network, showing relationships of “Chinese herbal-active ingredient-effector target-biological process-signaling pathway” (Fig. 8). In this network, 5 rectangles represent Chinese herbals, 18 triangles represent core potential active ingredients of XBJ, ellipses represent core top 10 potential effector targets of COVID-19, round rectangles represent core top 10 biological processes, hexagon represent core top 10 KEGG pathways, and octagons represent core top 10 Wikipathways. This indicated that the targets of the active ingredients of XBJ were distributed across different pathways and that XBJ might use multiple pathways to carry out its synergistic effects.

**Fig. 9:**
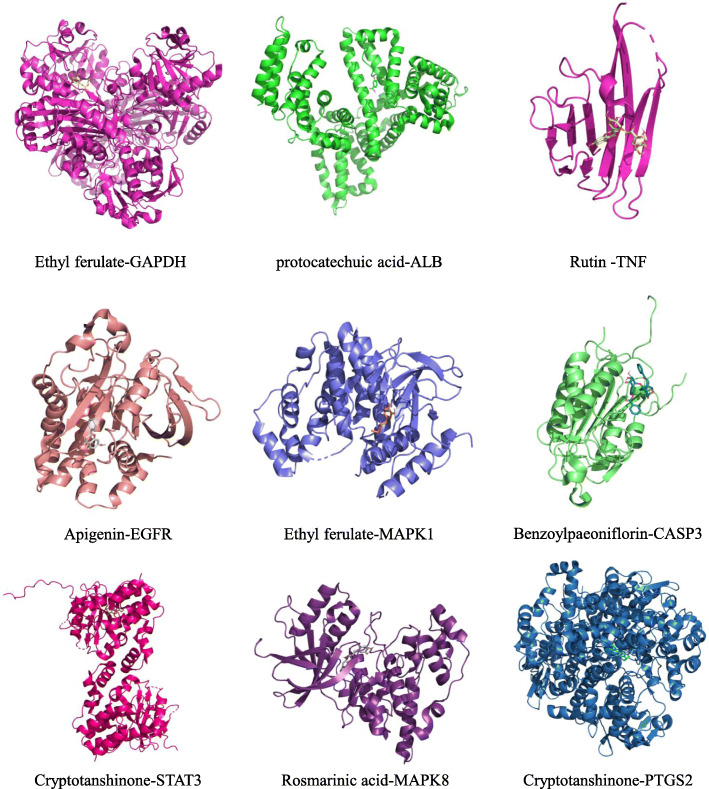
Binding modes of compounds and targets

We applied molecular docking to assess protein-ligand binding potential, top 10 potential effector targets and their related 18 ingredients in XBJ and were selected. The molecular docking results showed that the 18 effective ingredients had a docking score of less than − 4.0 with those 10 targets, indicating they have potential binding activity. Among them, Ethyl ferulate-GAPDH, protocatechuic acid-ALB, Rutin-TNF, Apigenin-EGFR, Ethyl ferulate-MAPK1, Benzoylpaeoniflorin-CASP3, Cryptotanshinone-STAT3, Rosmarinic acid-MAPK8, Cryptotanshinone-PTGS2 and Salvianolic acid B-JUN had best binding activity than others (Table 3). Nine binding modes are displayed in Fig. 9. Therefore, these ingredients may be potential compounds in anti-COVID-19 in XBJ.

**Table 3:** Top ten targets with their source composition information and docking score

No	Target (PDB-ID)	Degree	Molecule Name	Docking Score	No	Target (PDB-ID)	Degree	Molecule Name	Docking Score
1	GAPDH (6YNE)	77	Ethyl ferulate	−4.8	6	CASP3 (5IBP)	58	Salvianolic acid B	−6
2	ALB (1N5U)	73	protocatechuic acid	−5.8				Benzoylpaeoniflorin	−7.7
3	TNF (5UUI)	70	Galuteolin	−7.2				Rosmarinic acid	−5.5
			Rosmarinic acid	−7.3				Ethyl ferulate	−5.2
			Hyperoside	−5.5				Chlorogenic acid	−6.3
			Rutin	−7.9	7	STAT3 (6SM8)	55	Cryptotanshinone	−9.3
4	EGFR (3IZ7)	63	Tanshinol	−5.1				Caffeic acid	−6.6
			Caffeic acid	−5.5				Ethyl ferulate	−6.7
			Albiflorin	−6.5				Ferulic acid	−6.6
			Galuteolin	−6.9	8	MAPK8 (4QTD)	49	Rosmarinic acid	−4.7
			Rosmarinic acid	−5.3	9	PTGS2 (5IKT)	48	Cryptotanshinone	−9
			Ethyl ferulate	−5.4				Butylidenephthalide	−8
			Quercetin	−7.1				Galuteolin	−8.3
			Ferulic acid	−5.5				Hyperoside	− 8.8
			Luteolin	−7.1				Rutin	−8.7
			Apigenin	−7.2				Ferulic acid	−6.7
								Luteolin	−8.4
5	MAPK1 (6RFO)	61	Caffeic acid	−6.3				Apigenin	−8.6
			Ethyl ferulate	−5.9	10	JUN (6I0J)	47	Salvianolic acid B	−6.9

## Discussion

XBJ has been approved for the treatment of severe infection (sepsis). For a long period of time in China, it was believed that XBJ could improve prognosis in severe lung infection [35] as well as 28-day mortality rate, Acute Physiologic Assessment and Chronic Health Evaluation (APACHE) II score, white blood cell (WBC) count, and temperature in sepsis patients without causing serious adverse events. A prospective, randomized controlled trial in 33 hospitals in China, published in September 2019, showed that XBJ can significantly improve the primary endpoint of pneumonia severity in patients with severe community-acquired pneumonia, as well as secondary endpoints such as mortality rate, mechanical-ventilation duration, and length of intensive-care unit (ICU) stay [36]. Since the start of the COVID-19 outbreak, XBJ has been recommended as a Chinese patent medicine in local COVID-19 diagnosis and treatment plans released by many provincial health commissions due to its rapid onset and significant efficacy in critically ill patients. This shows that XBJ might have important clinical value in COVID-19 treatment. However, the material bases and molecular effector mechanisms are still unclear. Therefore, analysis of the potential effector mechanisms of XBJ in the treatment of COVID-19, elucidating its potential active ingredients and their potential effector targets, and demonstrating the network effector mechanisms of XBJ on COVID-19, can provide a scientific basis for using XBJ in the clinical treatment of COVID-19.

This study preliminarily demonstrated XBJ’s “multiple component-multiple target-multiple pathway” effector characteristics, and our network topology analysis of potential effector targets identified some critical effector targets of the compound. GO and KEGG enrichment analyses found that the potential targets of XBJ involved multiple inflammation- and immune-related gene functions and signaling pathways, which might be one basis for XBJ treatment in COVID-19.

From a potential active-ingredient perspective, XBJ possesses potential anti-inflammatory and immune-boosting effects [37]. The three active ingredients of *Carthamus tinctorius* have protective effects in lipopolysaccharide (LPS)-induced acute lung injury (ALI) [38]. The potential anti-inflammatory components in XBJ inhibit NF-κB activity; decrease expression of TNF-α, IL-1β, and IL-6 [39]; alleviate inflammatory responses; and inhibit secretion of pro-inflammatory cytokines mediated by the high-mobility group box 1 protein (*HMGB1*)-receptor for advanced glycation endproducts (*RAGE*) axis, thereby decreasing mortality rate in a mouse model [40]. XBJ also upregulates toll-interacting proteins in septic rats to protect the lungs from permeable leakage and injury [41]. In addition, it prevents cytokine storm, inhibits inflammatory responses, and regulates regulatory T-cell (Treg)-T helper 17 cell (T_h_17) balance to improve survival in septic shock [42]. In addition, XBJ promotes the expression of annexin A1 to inhibit cleavage of pro-inflammatory cytokines and decreases IL-8 and TNF-α levels to protect rats from damage due to *Acinetobacter baumannii* sepsis [43]. SARS-CoV-2 replicates in respiratory-tract epithelial cells to cause acute inflammation and severe respiratory disease. During infection, local production of pro-inflammatory cytokines exacerbates disease progression. Therefore, the anti-inflammatory activity and cytokine-inhibitory effects of XBJ might constitute its potential mechanism in COVID-19 treatment.

In GO BP analysis, the 144 potential therapeutic targets of XBJ involved multiple inflammation- and immune-related BPs such as extracellular signal-regulated kinase 1 and 2 (*ERK1*, *ERK2*) cascade, the T-cell receptor signaling pathway, activation of *MAPK* activity, and cellular response to LPS. This suggests that the significant anti-inflammatory effects of XBJ are its therapeutic effects in inflammation. Virus-host interactions are an important aspect of viral replication. Ribonucleic acid (RNA) viruses such as influenza, Ebola virus, and SARS-CoV can induce *Raf*–mitogen-activated protein kinase kinase (*MEK*)–*ERK* signal transduction in the *MAPK* cascade, which is associated with replication of pathogenic RNA viruses in humans and allows for cell differentiation and proliferation [44]. This is consistent with the fact that XBJ targets the *ERK1*/*ERK2* cascade.

Extensive proteinoid and serous exudates are present in the alveoli of COVID-19 patients. These cases also present bilateral diffuse alveolar damage accompanied by fibromyxoid exudates, and both lungs show apparent pulmonary edema, alveolar epithelial detachment, and hyaline-membrane formation [45]. In terms of infection-related serum markers, studies show that C-reactive protein (CRP), IL-6, and erythrocyte sedimentation rate (ESR) are significantly increased in many patients [46]. Severe cytokine storm can appear in severely to critically ill patients, resulting in excessive immune activation and excess production of IL-7, IL-10, granulocyte colony-stimulating factor (GCSF), interferon gamma inducible protein 10 kD (IP-10), monocyte chemoattractant protein 1 (MCP-1), macrophage inflammatory protein 1A (MIP1A), and TNF-α [47]. From this, it can be seen that SARS-CoV-2–mediated inflammation plays an important role in COVID-19 progression, and uncontrollable lung inflammation may be the main cause of death in COVID-19. Therefore, intervention measures to reduce inflammation might help decrease the mortality rate [48].

With regard to effector targets, the degree values of *GAPDH*, albumin (*ALB*), *TNF*, *EGFR*, *MAPK1*, *CASP3*, *STAT3*, *MAPK8*, *PTGS2*, *JUN*, *IL-2*, Estrogen Receptor 1 (*ESR1*), and *MAPK14* were all > 40, suggesting that in COVID-19 these could be the main therapeutic targets of XBJ’s active ingredients. Then we verified the binding activity of the XBJ key compounds and the COVID-19 key targets via molecular docking. The results indicated that they have potential binding activity and these ingredients may be potential compounds in anti-COVID-19 of XBJ. Wikipathways and KEGG enrichment analysis contained 40 and 94 signaling pathways, showing that the 144 potential XBJ therapeutic targets were involved in many inflammation- and immune-related signaling pathways.

The renin-angiotensin system (RAS), OS and cell death, cytokine storm, and endothelial dysfunction are four main pathways in COVID-19 pathogenesis. ACE is a receptor in the airway, alveoli, and vascular endothelium. COVID-19 uses ACE to enter type II pneumocytes or intestinal epithelial cells in order to induce ACE2 internalization and shedding, resulting in the occurrence and development of acute respiratory distress syndrome (ARDS) [49]. Many of XBJ’s active ingredients act on multiple targets in the RAS, which could potentially interfere with ACE receptors. NF-κB activation exacerbates lung inflammation caused by SARS-CoV infection, and inhibition of NF-κB signaling significantly reduces such inflammation and increases the survival rate of SARS-CoV-infected mice [50, 51]. In addition, NF-κB is an important transcription factor that induces expression of viral genes, and inhibition of NF-κB activation is an immune evasion mechanism of SARS-CoV [52]. Therefore, we speculate that XBJ’s active ingredients might interfere with the NF-κB pathway and regulate innate immunity and inflammation during viral infection to alleviate lung inflammation during COVID-19.

One study has shown that XBJ inhibits *MAPK* and NF-κB expression and has protective effects in ALI [53]. The compound regulates the NF-κB, *MAPK*, and *PI3K*–*Akt* pathways in mouse macrophages and downregulates inflammatory cytokines such as IL-6, TNF-α, MCP-1, MIP-2, and serum IL-10 to increase the survival rate of septic mice [15]. XBJ downregulates toll-like receptor 4 (*TLR-4*) and NF-κB expression to carry out its anti-inflammatory effects. *MAPK* activation can promote the expression and release of pro-inflammatory cytokines such as TNF-α and IL-1β, − 6, and − 8; it is a core factor in inflammation regulation. Viruses usually directly or indirectly affect the *PI3K*–*Akt* pathway to control intracellular-signaling pathways. EGFR aggregation and binding of influenza virus to cell surfaces might activate *Akt*. The *PI3K*–*Akt* signaling pathway might synergize with the RAS to promote viral entry, which has significant effects in viral infection in humans [54]. TLR-2, TLR-3, and TLR-4 activation by COVID-19 causes the release of inflammatory cytokines such as IL-1β. The binding of SARS-CoV-2 to TLRs causes release of pro-IL-1β, inflammasome activation, and production of mature IL-1β. Pro-inflammatory cytokines are important mediators of local and systemic inflammation. Viral particles first invade the respiratory mucosa before infecting other cells, thereby inducing a series of immune responses leading to cytokine storm [55]. Therefore, XBJ could be used to treat COVID-19 patients due to its anti-inflammatory effects, anti-immune apoptosis, and alleviation of pneumonia-induced multi-organ damage. In addition, we found that critical nodes on our “XBJ active-compound-potential effector target” network analysis map participated in the aforementioned pathways, suggesting that the predictions made in this study are somewhat accurate.

When compared to other research works on NP related to COVID-19 prevention and treatment, some of our research results and conclusions are consistent with the current similar research in some aspects. Qingfei Paidu Decoction (QFPD), a clinically used Chinese medicine for treating COVID-19 patients in China, has been shown by a recent NP research that the therapeutic effects of QFPD against COVID-19 may be attributed to the anti-inflammatory effects related to the thrombin and TLR signaling pathway [56]. What’s more, QFPD could protect COVID-19 injury via anti-Viral, anti-Inflammatory activity and metabolic programming [57]. Another research shows that Pudilan (PDL), clinically used as an anti-SARS-CoV-2 infective agent in China. PDL might moderate the immune system to shorten the course of the disease, delay disease progression, and reduce the mortality rate [58]. Hence, QFPD and PDL, together with XBJ may have a therapeutic effect on COVID-19 through three aspects, including the immune system, anti-inflammation, and anti-virus entry into cells.

Previous similar study have shown that 8 key compounds and 15 key targets of XBJ were screened and verified by molecular docking [59], on the basis of this research, we have made improvements in the criteria for screening active ingredients, drug targets, and disease targets (Table 4). As XBJ is administered intravenously, which does not need to be absorbed through the gastrointestinal tract, therefore, we selected active ingredients of XBJ that had been detected by LC-MS in our study. What’s more, we used the latest update version of Swiss database to predict the targets of XBJ. In terms of the potential core targets of COVID-19, we finally found different results from previous studies.

**Table 4:** The comparison and advantage of this research work with the recently published paper in the Traditional Medicine Research journal

Study	Compounds of XBJ^a^	Targets of XBJ^b^	Potential targets of COVID-19	Results	Software and tools	Combine with other targets^c^
Zhang et al	Based on TCMSP database (2014 version) with oral bioavailability (OB) ≥ 30% and drug-likeness (DL) ≥ 0.18.	TCMSP database.	Based on the GeneCards database.	8 key compounds: Luteolin, Quercetin, Baicalein, Kaempferol, Tanshinone II A, Myricanone, Dan-shexinkum d, Ellagic acid. 15 key targets: DPP4, AR, ESR1, CALM1, AKT1, CASP3, NOS3, VEGF-A, TP53, BCL2, TNF, JUN, CDKN1A, FOS and BAX.	Topology analysis: Network Analyzer. Network construction: Cytoscape. Enrichment analysis: Metascape, DAVID database. Molecular docking: Ligand Docking module of Schrödinger.	NO.
The current research	Selected active ingredients that had been detected by liquid chromatography-mass spectrometry (LC-MS).	Swiss database.	On the basis of Zhang’s research, we added two ways to search for disease targets, including literature search and ACE co-expressed genes.	18 key compounds were showed in Fig. 8. 10 key targets: GAPDH, ALB, TNF, EGFR, MAPK1, CASP3, STAT3, MAPK8, PTGS2, JUN, IL-2, ESR1, and MAPK14.	Topology analysis: STRING and cytoHubba. Network construction: Cytoscape. Enrichment analysis: R software 3.5.2, org.Hs.eg.db package, clusterProfiler package, and ClueGO. Molecular docking: Auto Dock vina, AutoDockTools, Protein Data Bank, and PyMOL.	YES. Combine with other targets like putative COVID-19-interacting human proteins.

^a^OB is an index used to screen active ingredients administered orally and is not suitable for intravenous injections, while XBJ is administered intravenously. OB and DL are often used to screen the effective ingredients of Chinese medicine compounds administered by oral route, the injection do not need to be absorbed through the gastrointestinal tract

^b^The latest update time of TCMSP was 2014, it is questionable whether overly lagging knowledge can facilitate mechanistic investigations of TCM against COVID-19. So, we used Swiss database (on-line since 2014, and the latest update time was 2019) to predict the targets of XBJ

^c^All 332 virus-interacting human proteins were obtained from STRING (https://string-db.org/cgi/covid.pl)

Compared with previous similar study, there is a significant advantage of our study. To further verify our analysis results, we found that the 451 targets of XBJ (Supplementary file, Table S1) and 332 putative COVID-19-interacting human protein had fourteen intersections: BRD2, BRD4, IMPDH2, POLA1, ATP6AP1, SIGMAR1, HMOX1, DNMT1, HDAC2, PABPC1, EIF4H, PRKACA, ABCC1, and COMT.

**Fig. 10:**
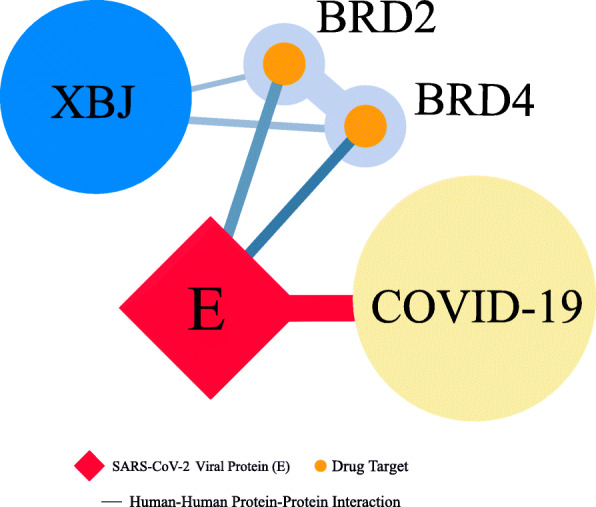
Potential mechanism of XBJ on COVID-19 by affecting the interaction between protein E and BRDs

It should be noted that BRD2/BRD4 are potential drug targets of Envelope (E) protein, a structural proteins of COVID-19, which plays a central role in virus morphogenesis and assembly. BRD2/BRD4 (Bromodomain extra terminal proteins) are implicated as epigenetic factors that regulate genes crucial for cell cycle progression, inflammation and immune response. What’s more, E binds to protein M, and co-expression of M and E is sufficient for VLP formation and release. Lack of E reduces viral titers about 20-fold [14]. From this speculation, XBJ might help the treatment of COVID-19 by disrupting the interaction between protein E and BRDs (Fig. 10).

However, there are several limitations in the current study. The host proteins and existing targets of the relevant coronaviruses are used to be potential therapeutic targets for COVID-19. Although SARS-CoV-2 shared high nucleotide sequence identity with other HCoVs, our predictions are not SARS-CoV-2 specific by lack of the known host proteins on SARS-CoV-2. In terms of COVID-19 treatment targets, we have an adopted alternative strategy that is currently achievable.

SARS is associated with epithelial-cell proliferation and an increase in macrophages in the lung [60]. And diffuse alveolar damage was seen in COVID-19 cases [61]. Regarding the differences between COVID-19 and SARS. Studies reveal that SARS-CoV-2 is very similar in structure and pathogenicity with SARS-CoV, but the most important structural protein, i.e., the spike protein (S), is slightly different in these viruses. Compared to other beta coronaviruses, the presence of a furin-like cleavage site in SARS-CoV-2 facilitates the S protein priming and might increase the efficiency of the spread of SARS-CoV-2 [62, 63]. COVID-19 has diverse epidemiological and biological characteristics, making it more contagious than SARS-CoV and MERS-CoV. It has affected more people in a short time period compared to SARS-CoV and MERS-CoV, although the fatality rate of MERS-CoV was higher than SARS-CoV and SARS-CoV [64].

We explore the molecular mechanism of Xuebijing injection in COVID-19 based on the premise of host and protein interaction. We use coronavirus-related host proteins as potential targets, which aim to produce an indirect intervention against viral targets for the treatment of COVID-19.

## Conclusions

XBJ could regulate different genes, act on different pathways, and synergize anti-inflammatory and immunoregulatory effects in COVID-19. This demonstrates the synergy of multiple targets and pathways among different components, as well as the holistic concept of TCM formulations.

## Supplementary information

**Additional file 1: Table S1.** The detailed targets information. **Table S2.** 451drug targets of XBJ. **Table S3.** 251 coronavirus pneumonia targets. **Table S4.** 119 HCoV-associated host proteins. **Table S5.** 3525 genes co-expressed with ACE2. **Table S6.** 144 potential COVID-19 targets. **Table S7.** Network topology analysis results. **Table S4.** 119 HCoV-associated host proteins. **Table S8.** GO-BP results. **Table S9.** KEGG-pathways results. **Table S10.** Wiki-pathways results.

## Data Availability

All data are available in the manuscript and are shown in tables, figures, and supplemental files.
